# Acute radiotoxicity studies and safety assessment of ^166^Ho-EDTMP and ^166^Ho-DOTMP for the palliative treatment of bone metastases

**DOI:** 10.1038/s41598-025-26098-7

**Published:** 2025-11-27

**Authors:** Ho Hong Quang Dang, Thi Khanh Giang Nguyen, Thi Ngoc Nguyen, Thanh Binh Nguyen, Ngoc Bao Nam Dinh, Ngoc Dieu Thao Le, Thu Minh Chau Nguyen, Hong Ngoc Quy Dang, Van Tien Le, Thanh Nhan Nguyen, Thi Thu Nguyen

**Affiliations:** 1 Center for Research and Production of Radioisotopes, Nuclear Research Institute, 01 Nguyen Tu Luc Street, Dalat City, Vietnam; 2 Department of Anapathology, Cho Ray Hospital, Ho Chi Minh City, Vietnam; 3 Hanoi Medical University, Hanoi , Vietnam; 4 Lam Dong General Hospital, Dalat City, Vietnam

**Keywords:** Biochemistry, Cancer, Cell biology, Molecular biology, Molecular medicine, Oncology

## Abstract

Comprehensive studies on the acute toxicity and safety profiles of therapeutic doses of ^166^Ho-EDTMP and ^166^Ho-DOTMP in the bone tissue and bone marrow of animals are limited, particularly when ^166^Ho produced in low-power reactors is used. These bone-specific complexes were synthesized through the coordination of ^166^Ho^3+^ ions in a neutral buffer. Acute toxicity studies were conducted at a dose of 1850 MBq/kg over 14 days, whereas safety assessments were performed at therapeutic doses of 18.5, 37, and 74 MBq/kg over 30 days in ICR mice. Hematological, biochemical, and histopathological toxicities were evaluated. ^166^Ho was obtained with high specific activities and radionuclide purity. These radiolabeled ligands were prepared with high radiochemical purity. No unexpected acute toxicity was observed. In the study groups, there were no significant signs of toxicity in the hematological, biochemical, or histopathological evaluations. However, a minimal reduction in osteocytes and osteoblasts in the bone marrow was noted. Notably, densely active hematopoiesis and progenitor clusters, including megakaryocytes, myeloblasts, and lymphoblasts, were observed in the bone marrow of the femurs and sternums. These findings provide valuable insights into the safety profiles of these radiolabeled ligands for bone and bone marrow, supporting further preclinical evaluations and domestic applications.

**Supplementary Information:**

The online version contains supplementary material available at 10.1038/s41598-025-26098-7.

## Introduction

 The incidence of cancer is steadily increasing. According to Globocan 2022, there are an estimated 20 million new cancer cases and 9.7 million cancer-related deaths worldwide^1^. Bone metastasis is a significant concern and occurs in 70–80% of all cancers, including breast, prostate, thyroid, lung, and kidney cancers; malignant melanoma; and head and neck, gastrointestinal, and ovarian cancers^2–4^. Bone metastases are often accompanied by pain due to complications such as bone destruction and cancer cell invasion^5^. The various treatment methods for bone metastases include chemotherapy, hormone therapy, radiotherapy, bisphosphonates, and pain-relief medications^6–8^. Radiopharmaceutical-based cancer treatment is a relatively new therapeutic approach that is gaining recognition and commercial acceptance^9^.

For pain relief in patients with bone metastases, radiopharmaceutical therapy that directly targets metastatic bone damage is effective and widely applied clinically. To reach these damaged areas, radionuclides need to be attached to phosphonates^2,10^. EDTMP (ethylene-diamine-tetramethylene phosphonic acid) and DOTMP (1,4,7,10-tetraazacyclododecane-1,4,7,10-tetramethylene phosphonate) are ligands that form coordination bonds with metal radioactive isotopes and localize on endosteal bone, where they are absorbed into the bone marrow endosteal^11,12^. To date, there are numerous reports on the use of ^166^Ho-EDTMP and ^166^Ho-DOTMP for treating pain from bone metastases and for bone marrow treatment in patients with multiple myelomas. Notable examples include studies by Bayouth et al., Joseph et al., and Ueno et al., as well as other clinical trials, such as NTC00039754 and NCT00008229^8,11–16^. In the past decade, several clinical reports have focused on radioactive drugs such as ^32^P, ^89^SrCl_2_, ^153^Sm-EDTMP, ^153^Sm-DOTMP, ^177^Lu-EDTMP, and ^166^Ho-DOTMP^11,16,17^. In addition, the radioactive beta-emitting isotopes used to treat bone pain include ^32^P, ^89^Sr, ^166^Ho, ^153^Sm, ^177^Lu, and ^143^Pr, along with alpha-emitting radioisotopes such as ^223^Ra, ^211^At, and ^225^Ac^10,17–19^.

^166^Ho is a radioactive isotope with many advantages due to its beta emission, with energies of 1.85 MeV (51%) and 1.77 MeV (48%). Additionally, ^166^Ho emits gamma rays with an energy of 80 keV (6.2%) and has a half-life of 26.8 h, making it suitable for imaging and treatment monitoring^8,13,20^. Furthermore, the emission range of β^−^ from ^166^Ho in tissues is 2 mm, with a maximum depth of 10.2 mm^21^. Despite their advantages, few commercial products are available; for example, ^166^Ho-Scout microspheres are used for liver cancer treatment in Europe, and ^166^Ho-Chitosan is approved for hepatocellular carcinoma therapy in Korea^16^. ^166^Ho forms stable complexes with EDTMP and DOTMP, showing high stability in both in vitro and in vivo studies^22,23^. Owing to its rapid concentration in bones, fast clearance from the blood, and minimal retention in soft tissues, its toxicity is minimal^15^. For the applications similar to ^153^Sm-EDTMP (Lexidronam), which has been FDA-approved since 1997 but is difficult to import due to its 1.9-day half-life and high cost^11^, the domestic production of ^166^Ho-EDTMP and ^166^Ho-DOTMP is appropriate and beneficial for local patients as a complementary alternative. In addition, ^166^Ho’s higher energy (E_βmean_ 0.66 MeV vs. 0.22 MeV for ^153^Sm) enhances therapeutic efficacy, requiring lower doses compared to ^153^Sm, which may benefit patients needing repeated treatments^21^. Besides, ^89^SrCl_2_ (approved as Metastron) has also been widely used for bone pain palliation, demonstrating clinical pain response rates comparable to ^153^Sm-EDTMP^20^. On the other hand, ^166^Ho-based radiopharmaceuticals provide better imaging quality in SPECT because it is within the optimal energy range for gamma camera detection, has less scatter, and allows for better dosimetry calculations^24^. ^166^Ho can be produced in a moderate-flux research reactor, whereas ^153^Sm requires a high-flux reactor, making labelled ^166^Ho more accessible in countries without high-flux neutron sources^21^.

While many clinical studies have been conducted on these labeled compounds in humans, a comprehensive preclinical safety evaluation in animals is lacking. To support regulatory drug registration, in this study, we meticulously investigated the production method of ^166^Ho using a local nuclear reactor, labeled it with ligands to prepare ^166^Ho-EDTMP and ^166^Ho-DOTMP, and performed quality control and preclinical evaluations, including assessments of acute radiotoxicity and safety assessment in animal tissues and bone marrow.

## Results

**Characterization of** ^**166**^**Ho**, ^**166**^**Ho-EDTMP**,** and** ^**166**^**Ho-DOTMP**.

**Fig. 1:**
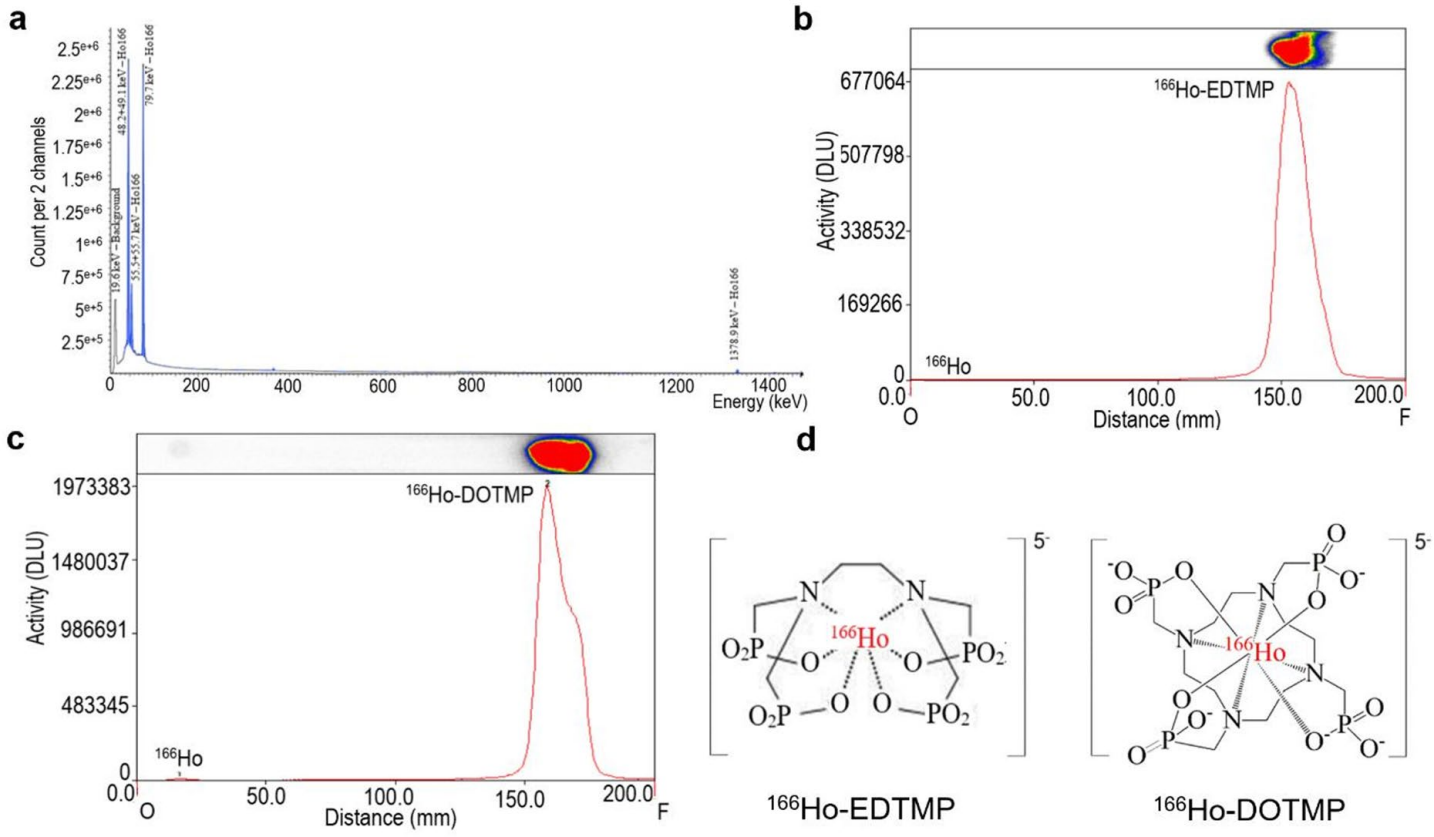
Characterization of ^166^Ho, ^166^Ho-EDTMP, and ^166^Ho-DOTMP. (**a)** Energy spectrum of ^166^Ho, gamma photons with 80 keV and 1379 keV, and X-rays with 48.2, 49.1 and 55.48 keV (low-energy gamma). **(b**,** c**) Radiochemical purities of ^166^Ho-EDTMP and ^166^Ho-DOTMP were determined via PC using Whatman No. 1 paper as the stationary phase and ammonia: methanol: water (1:10:20, v/v/v) as the mobile phase, and radioautography. Chromatograms were analyzed via OptiQuant 5 software; ^166^Ho remained at the origin, R_f_ = 0.0–0.25, and ^166^Ho-EDTMP and ^166^Ho-DOTMP migrated to the front, R_f_ = 0.75–1.00. (**d**) Hypothetical chemical structures of ^166^Ho-EDTMP and ^166^Ho-DOTMP.

The ^166^Ho radionuclide was produced in-house as ^166^HoCl_3_. The spectra revealed gamma emission at 80.6 keV (6.2%) and 1379.4 keV (0.9%), along with X-rays at 48.2 keV (2.8%) and 49.1 keV (5.0%)^13^. ^166^Ho emits high-energy beta particles with maximum energies (E_βmax_) of 1854 keV and 1774 keV, making it suitable for radionuclide therapy^16^, as shown in Fig. 1a. The radionuclide purity of ^166^Ho exceeded 99.9%. The radioactivity of the obtained ^166^HoCl_3_ solution was 19.70 ± 1.44 GBq/mL (0.264 mM/mL) using a Capintec ISOMED 2000 (USA). The specific activity of ^166^HoCl_3_ was 451.44 ± 33.53 MBq/mg, with a radiochemical purity of 99.62 ± 0.34 at calibration and experimentation (Table 1 and Supplementary Fig. S1a&b). EDTMP and DOTMP were labeled with ^166^Ho under optimized conditions^21,22^, including a molar ratio of 20:1 for EDTMP and 30:1 for DOTMP, pH 7.0, a 15-minute reaction time, and room temperature, which are presented in Supplementary Table S1. The radiochemical purities of ^166^Ho-EDTMP (Fig. 1b) and ^166^Ho-DOTMP (Fig. 1c) were greater than 98%. Both complexes demonstrated stability in 0.9% NaCl, 0.2 M phosphate buffer (pH 7.5), and 0.05% HSA, as shown in Fig. S1c&d. The EDTMP and DOTMP molecules contain four oxygen atoms in phosphonate groups and nitrogen atoms in amine groups, which participate in complexation with ^166^Ho^10^, as shown in Fig. 1d. Detailed characterization of the preparation batches revealed that the concentrations of ^166^Ho-EDTMP and ^166^Ho-DOTMP were 372.3 ± 2.3 MBq/mL and 374.6 ± 3.7 MBq/mL, respectively. Their radiochemical purities were 99.10 ± 0.55% and 99.35 ± 0.35%, with specific activities of 8.55 ± 0.62 and 4.53 ± 0.33 MBq/mg, respectively. At molar ratios of 1:20 and 1:30, together with mean efficacies of 99.10% and 99.35%, the average number of holmium atoms per EDTMP or DOTMP molecule was estimated to be 0.0495 and 0.0331, respectively, similar to the calculation method for ^90^Y/^131^I^25,26^, as shown in Table 1. This 3–5% ratio may reduce the risk of bone marrow toxicity from the high-energy beta emission of ^166^Ho. The abovementioned results confirm the high radiochemical purity, stability, and specific activity of radiolabeled ^166^Ho-EDTMP and ^166^Ho-DOTMP.

**Table 1:** Characteristics of ^166^Ho-EDTMP and ^166^Ho-DOTMP. The results are expressed as the means ± standard deviations; *n* = 3 for each experimental point (M/M: molar/molar ratio, mbq: Megabecquerel, RCP: radiochemical purity, SA: specific activity). (^a^) ^166^Ho^3+^ (mmol) = calculated as ~ 200 Mg Ho_2_O_3_ × 87.28% (Ho^3+^ in Ho_2_O_3_)/164.9 g.mol^−^, divided by 4 (volume of ^166^Ho^3+^ solution, 4 mL) = 0.264 mM (^b^) concentration: determined as the activity of ^166^HoCl_3_ (MBq)/mL (collected range: 74,275–82,880 MBq/4.0 mL). ^*^Radiochemical purity of ^166^Ho-EDTMP/^166^Ho-DOTMP was determined by a PC (using 20 × 200 mm strips). ^**^Specific activity (MBq/mg) of ^166^Ho: calculated as ^166^Ho activity/200 Mg ^165^Ho_2_O_3_ × 0.8728 Ho^3+^ content) = 451.44 ± 33.53. ^**^Specific activity (MBq/mg) of ^166^Ho-EDTMP: Range = 8.11–9.00. ^**^Specific activity (MBq/mg) of ^166^Ho-DOTMP: Range = 4.30–4.77. ^#166^Ho/EDTMP molar ratio: calculated as = 0.0132 mmol Ho^3+^/0.264 mmol EDTMP × 99.10% (efficiency) = 0.0495. ^#166^Ho/DOTMP molar ratio: calculated as 0.0132 mmol Ho^3+^/0.264 mmol DOTMP × 99.35% (efficiency) = 0.0331. The stability of ^166^Ho-EDTMP and ^166^DOTMP was evaluated under various conditions (Fig. S1a,b). A radiochemical purity exceeding 95% was observed at 4 °C and 24 °C in 0.9% NaCl and 0.2 M PBS and at 37 °C in 0.05% HSA after 3 days of storage.

Agents	mM^a^ (M/M)	Conc.^b^ (MBq/mL)	RCP^*^ (%)	SA^**^ (MBq/mg)	^166^Ho/Ligand^#^	Stability (%)
^166^HoCl_3_	0.264	19,701 ± 1,442	99.62 ± 0.34	451.44 ± 33.53	-	> 99.9
^166^Ho-EDTMP	20:1	372.3 ± 2.3	99.10 ± 0.55	8.55 ± 0.62	0.0495	> 95.0
^166^Ho-DOTMP	30:1	374.6 ± 3.7	99.35 ± 0.35	4.53 ± 0.33	0.0331	> 96.0

### Acute radiotoxicity results

#### Body weight changes

We administered a single dose of 37.0 MBq of either ^166^Ho-EDTMP or ^166^Ho-DOTMP to the mice. This dose corresponds to 100 times the normal therapeutic dose^27^, with specific activities of 9.02 MBq/mg and 4.77 MBq/mg, respectively. A significant weight increase was observed in the treated groups of both males and females, as shown in Supplementary Table S2. On Day 1, there were no significant differences in weight between the ^166^Ho-EDTMP- and ^166^Ho-DOTMP-injected groups (*P* > 0.05, row factor, *P* = 0.4274; ordinary two-way ANOVA). By Day 14, all the groups exhibited significant weight gain–with ^**^P < 0.01 and ^*^P < 0.05 in the control group and treated group, respectively–except for the female group injected with ^166^Ho-EDTMP (*P* = 0.0686, two-way ANOVA with Sidak’s multiple comparisons test)^28^.

## Hematological and biochemical analysis

**Fig. 2:**
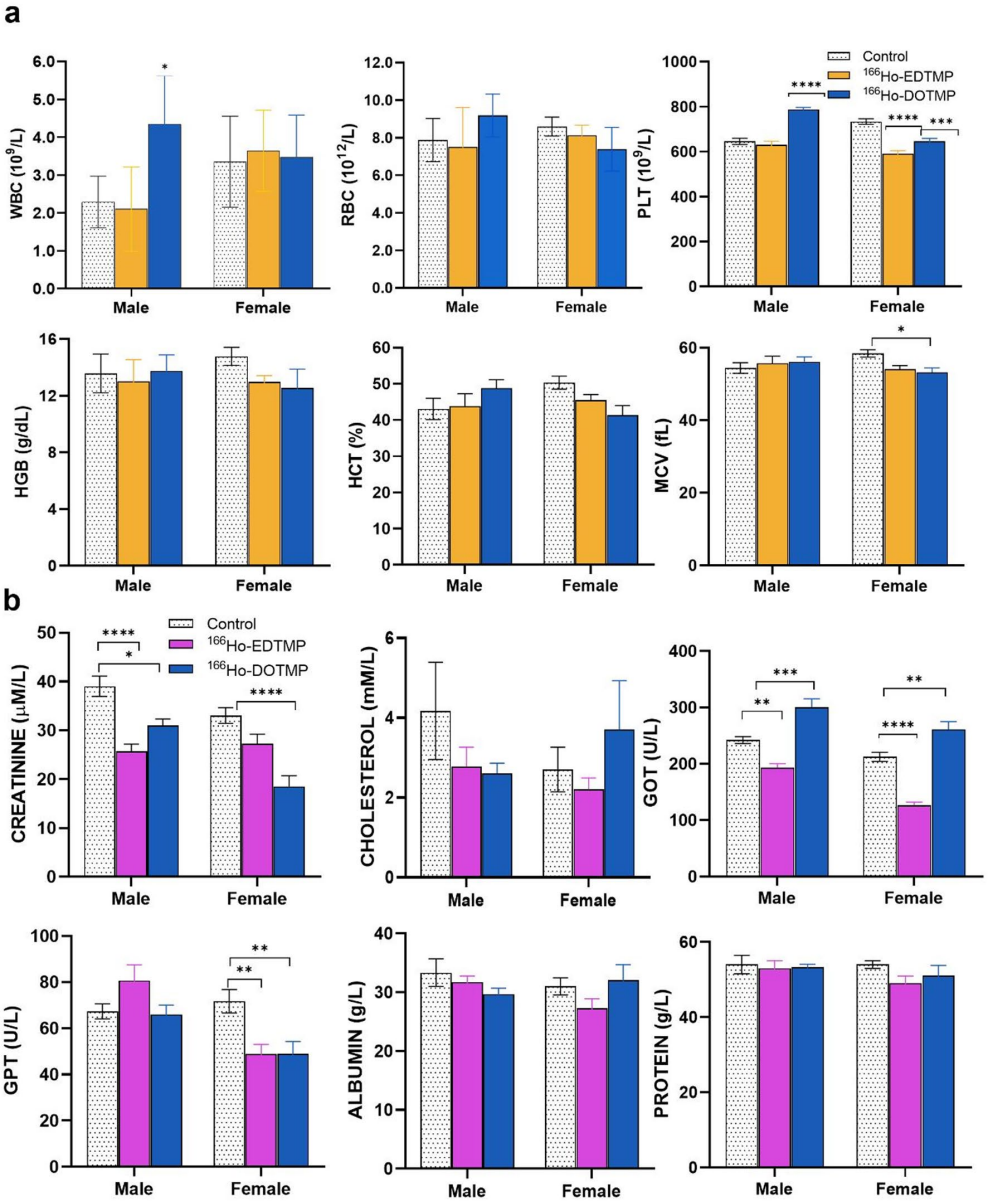
Hematological (**a**) and biochemical (**b**) analysis on Day 14 after the injection of 0 (0.9% NaCl) or 1850 MBq/kg ^166^Ho-EDTMP or ^166^Ho-DOTMP. The results are expressed as the mean ± SEM, with *n* = 4–5 for each group. The group marked with an asterisk (^*^) indicates a significant difference compared with the corresponding control group (*P* < 0.05).

In general, the hematological and biochemical parameters of the ^166^Ho-EDTMP and ^166^Ho-DOTMP groups at 24 h postinjection (p.i.) did not differ significantly from those of the control groups (*P* > 0.05, two-way ANOVA). On Day 14, the platelet count (PLT) in the ^166^Ho-EDTMP and ^166^Ho-DOTMP female groups was significantly lower than that in the control female groups (row factor: *P* = 0.0089; column factor: *P* < 0.0001, two-way ANOVA). No significant differences were detected (*P* > 0.05) in cholesterol, albumin, or protein levels. A significant decrease in creatinine levels was noted in male and female mice injected with ^166^Ho-DOTMP on Day 14 (*P* = 0.0125 and *P* < 0.0001, respectively). Glutamic oxaloacetic transaminase (GOT) levels were significantly lower in the ^166^Ho-EDTMP group than in the control group (*P* = 0.0036), whereas the ^166^Ho-DOTMP group presented significantly higher levels than did the control group (*P* = 0.0007). Glutamic pyruvic transaminase (GPT) levels in the ^166^Ho-EDTMP and ^166^Ho-DOTMP female groups were significantly lower than those in the control female group (*P* = 0.0059 and *P* = 0.0065, respectively). The results are presented in Fig. 2 and Supplementary Table S3. These results may be associated with the mild hepatitis and tubulointerstitial nephritis observed in mice treated with ^166^Ho-EDTMP and ^166^Ho-DOTMP^29^.

### Histopathological analysis

In the treatment groups, no significant lesions, necrosis or malignancy were found in the mice injected with ^166^Ho-EDTMP or ^166^Ho-DOTMP.

Histological analysis of tissues from 60 mice on Day 1 revealed mild inflammatory changes in the livers of 4 out of 20 male mice in the ^166^Ho-EDTMP group. In the ^166^Ho-DOTMP group, mild reactive inflammation of liver tissues was observed in 4 out of 20 mice (3 males and 1 female). No abnormal histological findings were detected in the kidneys or spleen tissues, and no mice died during this period.

**Fig. 3:**
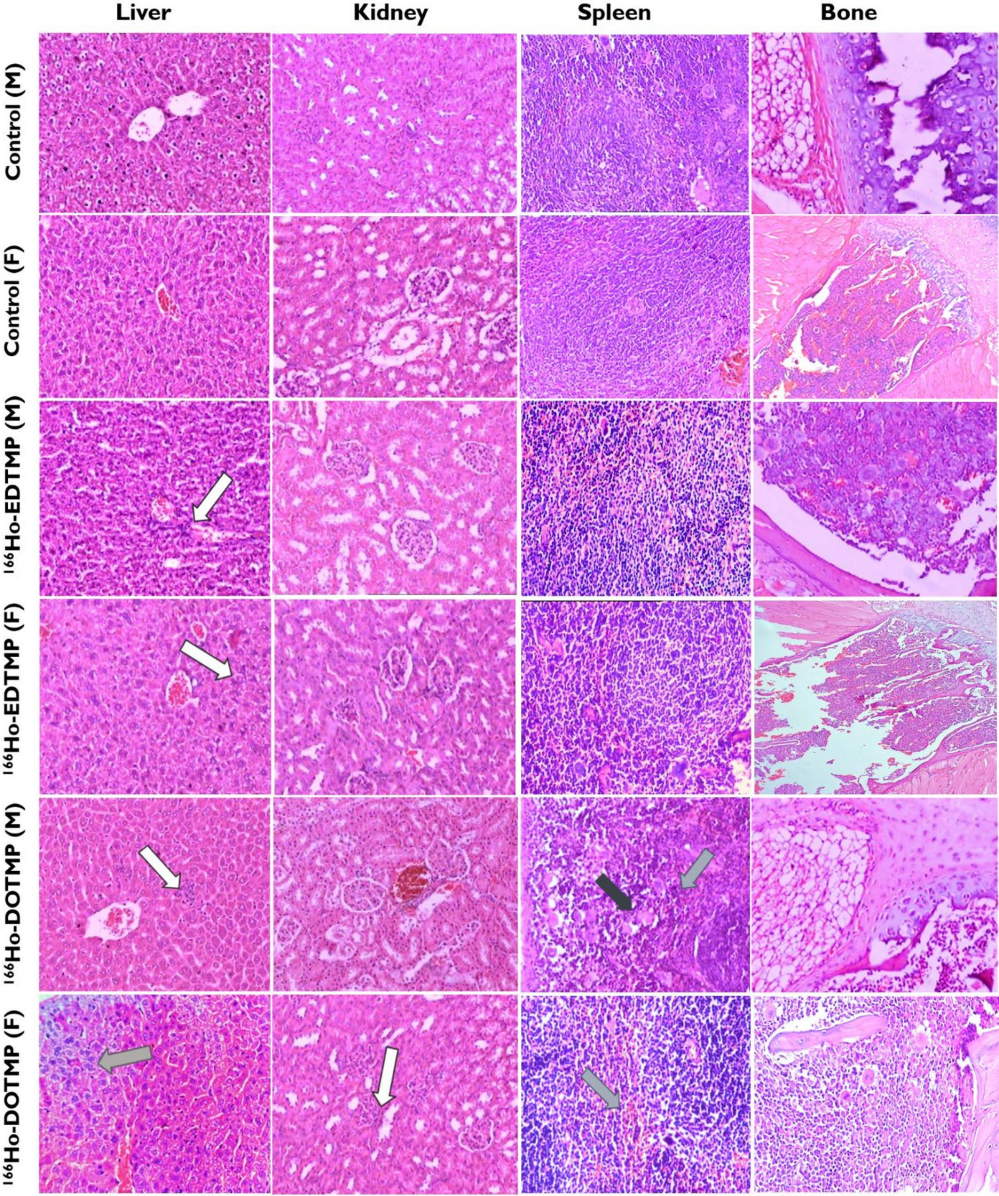
Microscopy images of the liver, kidney, spleen and femur tissues of the mice in all the groups (*n* = 5) observed on Day 14 after 0.9% NaCl, ^166^Ho-EDTMP and ^166^Ho-DOTMP injection. Tissues were stained with H&E and imaged (on an Olympus microscope, DP74, magnification 400×). White arrow: mild liver inflammation and mild internephritis area. Gray arrow: mild necrotizing hepatitis and hemorrhage. Black arrow: macrophages.

Histopathological examination of tissue samples from 30 mice on Day 14 revealed mild chronic hepatitis in 4 out of 10 mice (2 males and 2 females) in the ^166^Ho-EDTMP group. In the ^166^Ho-DOTMP group, 2 out of 10 mice (1 male and 1 female) exhibited tubulointerstitial nephritis. Hemorrhage and an increased density of macrophages were observed in the spleen of one female mouse treated with ^166^Ho-DOTMP (Fig. 3). Four mice, accounting for 4.4%, including 2 males injected with ^166^Ho-EDTMP and 1 male and 1 female injected with ^166^Ho-DOTMP, died during the experiment; however, no abnormalities were detected in their tissues. At a dose of 3.7 MBq per mouse, no histopathological differences were observed in the liver, kidneys, spleen, or femur across all groups during microscopic evaluation, except for mild hepatocyte inflammation and occasional hemorrhage in the kidneys and spleen in 1–2 mice on Day 14. Notably, no significant abnormalities were detected in the femurs.

### Safety profile of mice injected with ^166^Ho-EDTMP or ^166^Ho-DOTMP for 30 days

#### Clinical observations

Five mice, accounting for 7.1% of the study population, died. This included three mice injected with ^166^Ho-EDTMP (one male at 37 MBq/kg, and one female and one male at 74 MBq/kg) and two mice injected with ^166^Ho-DOTMP (one male at 37 MBq/kg and one female at 74 MBq/kg). All remaining mice appeared healthy, were agile, and ate and drank normally. There were no unusual changes in appearance, behavior, or locomotor activity, and no clinical signs of toxicity were observed.

### Body weight measurements

**Fig. 4:**
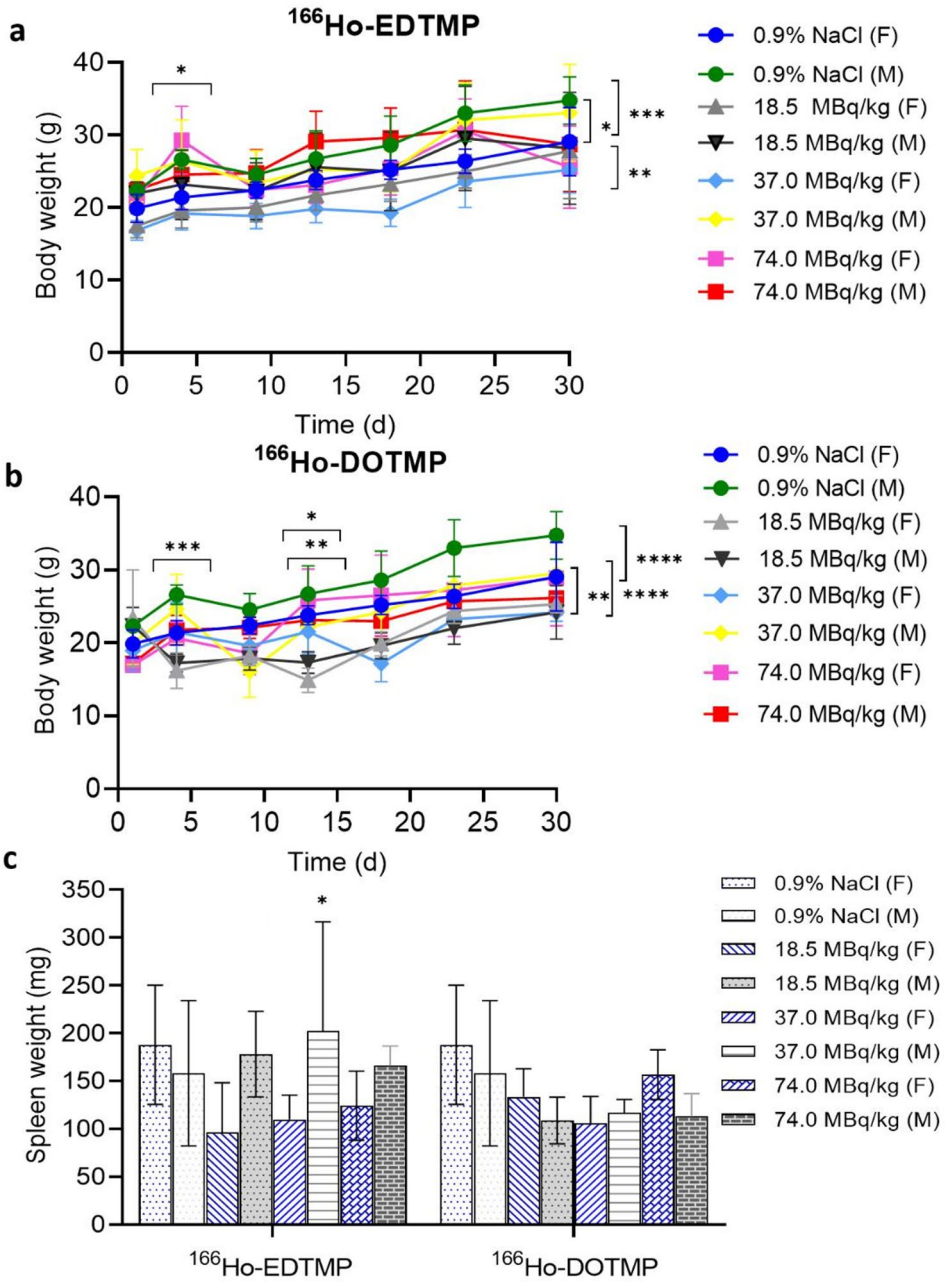
Body and spleen weight analysis of the mice on Day 30 after 0 (0.9% NaCl), 18.5, 37.0 and 74.0 MBq/kg ^166^Ho-EDTMP and ^166^Ho-DOTMP-injected doses, *n* = 4–5, mean ± SD. (**a)** Body weights of the mice in the 0.9% NaCl and ^166^Ho-EDTMP-injected groups. There were no significant differences in weight (*P* = 0.7255, two-way ANOVA with Tukey’s multiple comparisons test). (**b**) Body weights of the mice in the 0.9% NaCl and ^166^Ho-DOTMP-injected groups. There were significant differences in weight decrease (*P* < 0.0001, two-way ANOVA). (**c**) Spleen weights of the mice in the 0.9% NaCl, ^166^Ho-EDTMP and ^166^Ho-DOTMP-injected groups. There were no significant differences in spleen weight (*P* = 0.4010, two-way ANOVA).

For the female groups injected with ^166^Ho-EDTMP, the initial body weights were recorded as follows: 19.88 ± 1.93 g (0.9% NaCl), 17.50 ± 1.67 g (18.5 MBq/kg), 16.84 ± 1.35 g (37 MBq/kg), and 21.16 ± 1.48 g (74 MBq/kg). On Day 30, the weights were 29.08 ± 4.72 g, 27.80 ± 6.57 g, 25.20 ± 3.19 g, and 25.57 ± 5.67 g, respectively. The difference in mean weight from the control group on Day 30 was not significant, except for the 37 MBq/kg group (*P* = 0.0015, two-way ANOVA). Similarly, for the male groups injected with ^166^Ho-EDTMP, the initial body weights were 22.30 ± 0.84 g, 21.96 ± 1.59 g, 24.40 ± 3.65 g, and 22.44 ± 1.76 g, respectively. On Day 30, the weights were 34.77 ± 3.26 g, 28.17 ± 7.71 g, 33.06 ± 6.67 g, and 28.70 ± 6.51 g, respectively. The differences from the control group were generally insignificant, except for the 18.5 MBq ^166^Ho-EDTMP group (*P* = 0.0383, two-way ANOVA), which is shown in Fig. 4a.

For the female groups injected with ^166^Ho-DOTMP, the initial body weights were recorded as follows: 19.88 ± 1.93 g (0.9% NaCl), 23.36 ± 6.65 g (18.5 MBq/kg), 18.94 ± 2.81 g (37 MBq/kg), and 16.94 ± 0.94 g (74 MBq/kg). On Day 30, the weights were 29.08 ± 4.72 g, 25.32 ± 1.17 g, 24.15 ± 1.19 g, and 28.90 ± 6.55 g, respectively. The mean weight differences from those of the control group on Day 30 were not significant, except for those of the 18.5 MBq/kg group (*P* < 0.001, two-way ANOVA) and the 37 MBq/kg group (*P* = 0.0011, two-way ANOVA). Similarly, for the male groups treated with ^166^Ho-DOTMP, the initial body weights were 22.30 ± 0.84 g (0.9% NaCl), 22.36 ± 2.52 g (18.5 MBq/kg), 19.94 ± 2.88 g (37 MBq/kg), and 17.22 ± 0.47 g (74 MBq/kg). On Day 30, the weights were 34.77 ± 3.26 g, 24.30 ± 3.66 g, 29.54 ± 4.92 g, and 26.16 ± 2.30 g, respectively. Significant mean weight differences from those of the control group were observed in all treatment groups (*P* < 0.0001, two-way ANOVA), as shown in Fig. 4b.

### Spleen weight measurements

For the female groups injected with 0.9% NaCl and the three treatment groups, the spleen weights on Day 30 were 187 ± 62 mg, 117 ± 23 mg, 110 ± 25 mg, and 124 ± 36 mg (mean ± SD), respectively. The differences among the three treatment groups and the control female group were not significant, except for the NaCl group and the 37 MBq/kg ^166^Ho-EDTMP group, which presented a weight difference of 152.3 mg (*P* = 0.0213, two-way ANOVA). For the male groups injected with 0.9% NaCl and the three treatment groups, the initial spleen weights were 158 ± 75 mg, 108 ± 24 mg, 116 ± 14 mg, and 113 ± 23 mg (mean ± SD), respectively, as shown in Fig. 4c.

### Hematological and biochemical parameters

The hematological parameters of the groups injected with radioactive substances were not significantly different from those of the control groups (*P* > 0.05). However, an increase in the white blood cell (WBC) count was noted in the male group receiving 37 MBq/kg ^166^Ho-EDTMP and the female group receiving 74 MBq/kg ^166^Ho-DOTMP, despite the P value being 0.6979 (ordinary one-way ANOVA). There was a significant increase in hematocrit (HCT) levels in the female groups receiving 37 and 74 MBq/kg ^166^Ho-DOTMP (*P* = 0.0411 and 0.0351, two-way ANOVA). Additionally, the PLT was significantly reduced in the 74 MBq/kg ^166^Ho-DOTMP male group (^*^P = 0.0092, two-way ANOVA). The results are shown in Table 2. Although there was a mild increase in HCT and a slight decrease in thrombocytopenia, this increase was observed only in one group. The levels of biochemical parameters, including GOT and GPT, in the radioactive-injected groups were not significantly different from those in the control groups, with P values of 0.3322 and 0.3579, respectively.

**Table 2:** Hematological and biochemical analyses on day 30 after injection of 0 (0.9% NaCl), 18.5, 37.0 and 74.0 MBq/kg ^166^Ho-EDTMP and ^166^Ho-DOTMP. The results are expressed as the means ± SDs, *n* = 4–5 for each group. Notes: (#) indicates the normal ranges of murine hematological and biochemical indices on the basis of reference data from en.wikivet.net. Groups marked with an asterisk (*) are significantly different from the corresponding control group (*P* < 0.05).

Parameters	^166^Ho-EDTMP			^166^Ho-DOTMP	
(MBq/kg)	Female	Male	Female		Male
WBC (×10^9^/L) 2–10 (×10^9^/L)^#^					
0	4.83 ± 1.86	4.57 ± 3.48	4.83 ± 1.86		4.57 ± 3.48
18.5	6.14 ± 2.36	8.48 ± 1.24	7.79 ± 2.61		4.63 ± 1.25
37.0	5.67 ± 1.64	6.00 ± 2.18	5.97 ± 2.33		5.84 ± 2.83
74.0	4.04 ± 0.91	5.53 ± 2.62	7.58 ± 2.63		5.87 ± 1.34
RBC (×10^12^/L), 7.8–10.6 (×10^12^/L)					
0	9.15 ± 1.07	9.40 ± 0.39	9.15 ± 1.07		9.40 ± 0.39
18.5	9.00 ± 0.52	8.95 ± 0.64	9.29 ± 0.23		9.34 ± 0.48
37.0	8.93 ± 0.81	9.57 ± 0.63	9.67 ± 1.02		10.14 ± 0.96
74.0	9.37 ± 0.64	7.90 ± 4.31	9.63 ± 0.45		9.42 ± 0.66
HGB (g/dL), 10–16 (g/dL)^#^					
0	13.15 ± 1.26	14.38 ± 0.48	13.15 ± 1.26		14.38 ± 0.48
18.5	13.68 ± 0.64	14.45 ± 0.70	14.54 ± 0.21		14.48 ± 0.43
37.0	14.10 ± 0.95	14.26 ± 1.05	15.12 ± 1.18		15.13 ± 0.15
74.0	14.78 ± 1.00	11.94 ± 6.32	14.53 ± 0.68		15.06 ± 1.24
HCT (%), 39–49 (%)^#^					
0	45.00 ± 0.87	50.34 ± 2.03	45.00 ± 0.87		50.34 ± 2.03
18.5	47.96 ± 3.17	48.45 ± 3.04	51.18 ± 0.94		51.19 ± 3.76
37.0	48.78 ± 3.77	51.62 ± 4.03	52.78 ± 2.53^*^		52.88 ± 2.15
74.0	50.82 ± 3.86	52.53 ± 8.87	51.28 ± 2.61^*^		50.78 ± 3.03
PLT (×10^9^/L), 160–410 (×10^9^/L)^#^					
0	586 ± 140	716 ± 139	586 ± 140		716 ± 139
18.5	545 ± 124	609 ± 48	627 ± 44		606 ± 119
37.0	564 ± 137	558 ± 93	665 ± 104		621 ± 103
74.0	548 ± 36	441 ± 54	540 ± 119		501 ± 110^**^
GOT (U/L), 54–298 (U/L)^#^					
0	171.60 ± 59.32	197.40 ± 87.24	171.60 ± 59.32		197.40 ± 87.24
18.5	138.60 ± 31.48	152.00 ± 42.63	167.00 ± 42.11		203.50 ± 451.86
37.0	136.20 ± 20.72	155.20 ± 36.72	172.60 ± 48.81		177.00 ± 43.18
74.0	149.40 ± 39.23	186.85 ± 93.14	157.75 ± 43.00		169.60 ± 89.76
GPT (U/L), 17–77 (U/L)^#^					
0	60.80 ± 12.64	57.20 ± 11.54	60.80 ± 12.64		57.20 ± 11.54
18.5	46.60 ± 10.60	48.00 ± 4.24	45.80 ± 8.84		46.75 ± 13.55
37.0	43.20 ± 5.63	52.60 ± 8.44	57.60 ± 10.14		78.50 ± 46.54
74.0	44.40 ± 7.92^*^	56.58 ± 29.25	54.50 ± 11.62		51.60 ± 4.04

### Histopathological findings

**Fig. 5:**
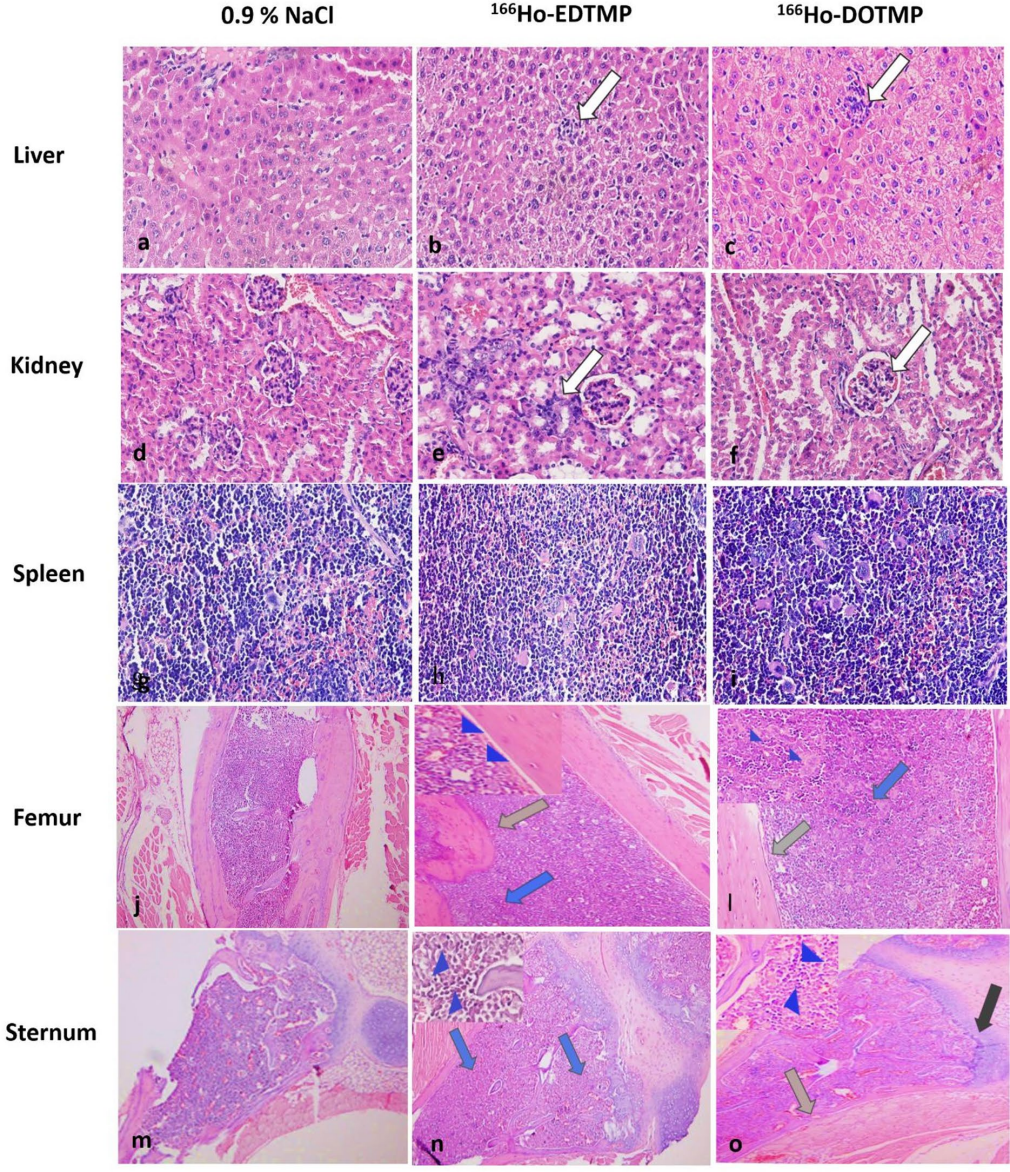
Microscopy images of tissues from control and study mice, highlighting observations made 30 days after injection. The liver, kidney, spleen and decalcified bone tissues were stained with H&E and imaged via an Olympus microscope (DP74, 100× and 400× magnification). White arrow: mild inflammation in tissues. Gray arrow: depletion of osteoblasts. The black arrow indicates osseous lesions. Blue arrow: At 100× magnification, the bone marrow is densely populated with hematopoietic cells. Blue arrowheads: At 400× magnification, dense hematopoietic cellularity in the bone marrow was observed. (**a**) Liver tissue of male mice in the control group injected with 0.9% NaCl. (**b**) Liver tissue of female mice injected with 37 MBq/kg EDTMP. (**c**) Liver tissue from a male mouse injected with 37 MBq/kg DOTMP. (**d**) Kidney tissue from a male injected with 0.9% NaCl. (**e**) Kidney tissue from a female injected with 74 MBq/kg EDTMP. (**f**) Kidney tissue from a male injected with 74 MBq/kg DOTMP. (**g**) Spleen tissue from a male injected with 0.9% NaCl. (**h**) Spleen tissue from a male injected with 74 MBq/kg EDTMP. (**i**) Female spleen tissue injected with 74 MBq/kg DOTMP. (**j**) Femur tissue from a male injected with 0.9% NaCl. (**k**) Femur tissue from a male injected with 74 MBq/kg EDTMP. (**l**) Femur tissue, female, injected with 74 MBq/kg DOTMP. (**m**) Sternum tissue, male, injected with 0.9% NaCl. (**n**) Sternum tissue, female, injected with 37 MBq/kg EDTMP. (**o**) Sternum tissue, male, injected with 74 MBq/kg DOTMP.

 Tissues were examined using hematoxylin and eosin (H&E) staining, a standard technique for pathological analysis^32^. Microphotographs of the tissues collected from the mice on Day 30 revealed that, concerning liver pathology, no significant lesions, necrosis, or malignancies were present in either treatment group. However, mild reactive inflammation was observed in 15 out of 58 mice (25.8%) across both sexes in the treated groups (Fig. 5b and c). Similarly, the kidneys showed no significant lesions, although mild tubulointerstitial nephritis was detected in 11 out of 58 mice (18.9%), as shown in Fig. 5e and f. Moreover, the spleen tissue appeared normal, with balanced red and white pulp regions and normal vascular distribution (Fig. 5h and i). These findings suggest a potential tissue response to the treatments, with the specific doses administered correlating with the observed mild effects. In terms of the pathohistology of the femurs and sternums on Day 30, minimal to mild depletion of osteocytes and osteoblasts in the femurs (13 mice) and sternums (5 mice) of the ^166^Ho-EDTMP group, and in the femurs (10 mice) and sternums (7 mice) of the ^166^Ho-DOTMP group. Additionally, a few instances of peri-trabecular fibrosis at minimal levels were observed in three and two mice, respectively, as summarized in Table S4**.** The osteocytes were mostly healthy within their lacunae, showing no vacuolization or pyknotic nuclei. Osteoblasts at 400× magnification, which are not prominent along the trabecular or cortical bone, reflect reduced bone remodeling activity (Fig. 5k, l). In addition, there was dense hematopoietic cellularity in the bone marrow, as shown in Fig. 5 with a gray arrow.

**Fig. 6:**
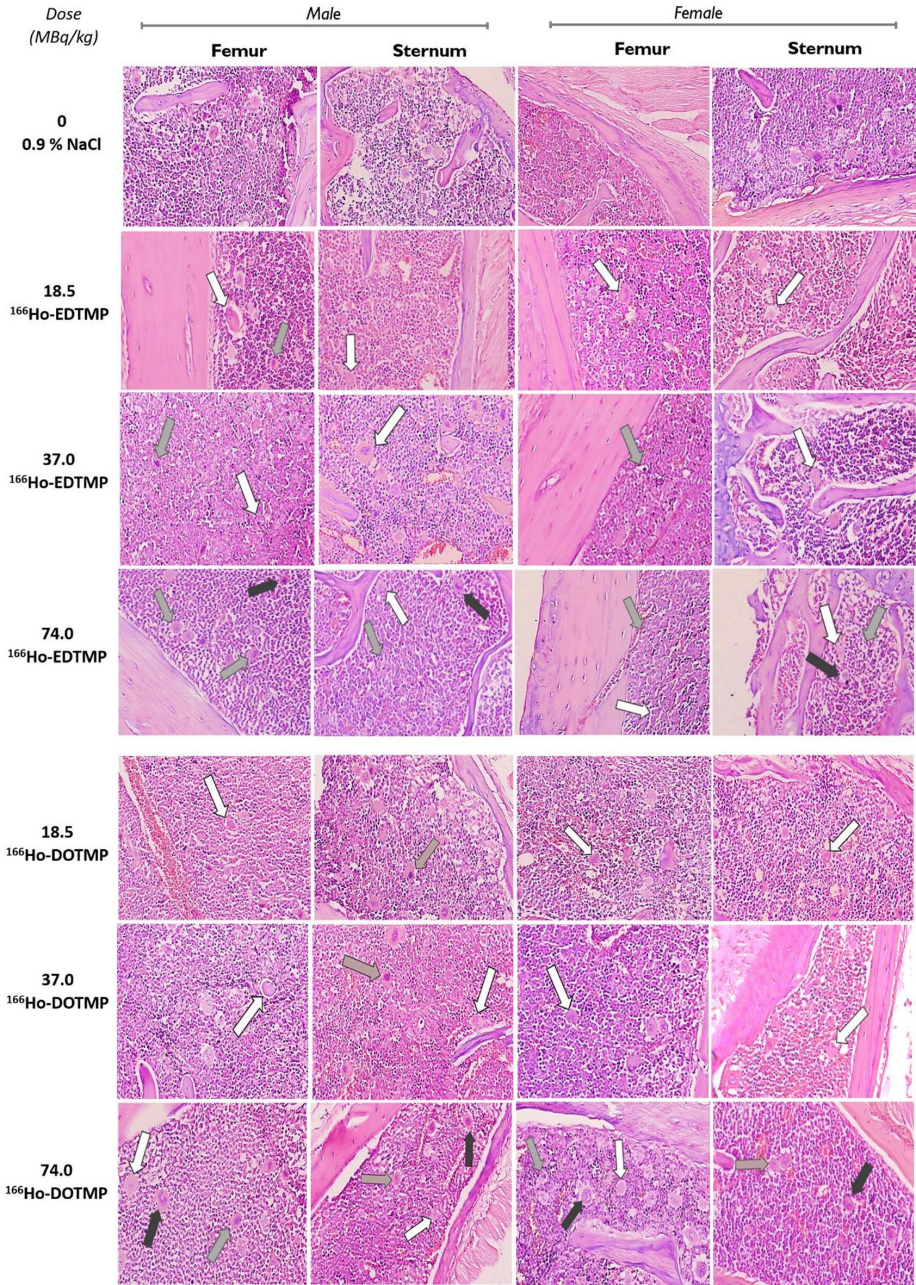
Microscopy images of the bone marrow from the femur and sternum, which represent bone tissues from the male and female groups (*n* = 5), injected with different doses of ^166^Ho-EDTMP and ^166^Ho-DOTMP. Observations were made on Day 30 postinjection, with the tissues decalcified, H&E stained, and photomicrographed using an Olympus DP74 microscope at 400× magnification. White arrow: Megakaryocyte, a large hematopoietic cell characterized by a multilobed nucleus. Gray arrow: Lymphoblast cell, identified by its 1–2 nucleoli, scant cytoplasm, and round or slightly irregular nucleus. Black arrow: Myeloblast cells, large hematopoietic precursor cells with scant cytoplasm, round or slightly irregular nuclei, fine chromatin, and 3–5 nucleoli.

In addition, the bone marrow in Fig. 6 shows active hematopoiesis, with no significant suppression or abnormalities. As shown in Supplementary Fig. S2, the sternal bone marrow was clearly identifiable and well preserved. The bone marrow areas exhibited large cells, with their density gradually increasing in correlation with the injection dose. Specifically, the percentages of these cells in the ^166^Ho-EDTMP-injected groups at doses of 18.5, 37, and 74 MBq/kg were 10%, 20%, and 30%, respectively. Similarly, the percentages of observed cells in the ^166^Ho-DOTMP-injected groups at doses of 18.5, 37, and 74 MBq were 10%, 15%, and 20%, respectively. These large cells may include megakaryocytes, lymphoblasts, and myeloblasts and are characterized by multilobulated nuclei containing 1–2 or 3–5 lobes, along with scant cytoplasm and round or slightly irregular shapes^33,34^. The cell nuclei appeared normal, without hyperchromasia or fragmentation, indicating an absence of radiation-induced apoptosis. Megakaryocytes were visible, suggesting preserved platelet precursor activity. No signs of infiltration by extramedullary cells were observed. Cancer metastases, osteoblastomas, osteofibromas, and osteosarcomas were not detected.

These findings suggest that while the bone marrow remains functional and healthy, there might be slight suppression of osteoblast activity, possibly due to the radiopharmaceutical’s impact on bone remodeling processes.

## Discussion

In this study, we demonstrated that the radiotoxic effects of ^166^Ho-EDTMP and ^166^Ho-DOTMP on hematotoxicity and histology, particularly in the pathohistological analysis of femurs and sternums in ICR mice, revealed no significant abnormalities, although further investigation is needed.

We produced ^166^Ho in-house to ensure availability and reliability, because of its short half-life of only 26.8 h and the challenges of importation. Using neutron activation in a low-power reactor at a flux of 2.3 × 10^13^n/cm²/s, which is similar to the flux studied by Bayouth et al. and Bahrami-Samani et al.^13,22^, we successfully prepared ^166^Ho with a reasonably high specific activity of up to 2.84 GBq/mg Ho^3+^, and a holmium concentration of 0.264 mM, meeting the requirement of 2.42 mM as per the authors^13,14^. ^166^Ho is a promising radionuclide for theranostic applications because of its high-energy beta emission, which provides excellent therapeutic efficacy, and gamma emission, which is suitable for diagnostic imaging^13–16^. Additionally, the beta particles from ^166^Ho have a penetration depth of approximately 2.2 mm in soft tissue, with over 90% of the radiation deposited within tissues^21^. This characteristic enables targeted radiation delivery to the bone and bone marrow while sparing normal tissues^15,16^. The bone marrow receives β⁻ irradiation mainly from trabecular bone regions such as the femur, tibia, humerus, and skull, confirming that marrow dose primarily originates from bone-associated sources^35^. Liganded ^166^Ho emits β⁻ radiation delivering the highest dose within ~ 2 mm from the bone surface, decreasing at ~ 10 mm (~ 200–1000 cells)^36^. Owing to its short half-life governed by decay law N(t) = N₀e − λt, ^166^Ho limits radiation exposure to the bone marrow. Moreover, pain palliation efficacy depends not only on β⁻ penetration depth but also on energy deposition, biodistribution, and bone affinity. For example,^166^Ho, ^188^Re, ^89^Sr, ^153^Sm, and ^177^Lu, with β⁻ ranges of 10, 11, 8, 3, and 2 mm, have shown clinical pain response rates of 35–91%, 70–80%, 60–95%, 62–74%, and 77–95%, respectively^8,10,16,37^. Nevertheless, the potential risks should also be considered, including transient myelosuppression (reduced WBC or PLT counts) and localized dose heterogeneity within trabecular bone that may cause focal marrow exposure. The alternative radionuclides with lower β⁻ energy, such as ^153^Sm-EDTMP, ^89^Sr, or ^177^Lu-EDTMP, may offer safer and effective options for bone pain palliation, as summarized in Supplementary Table S5. The primary reason for selecting EDTMP and DOTMP in this study is their high affinity for inorganic salts in bones, such as calcium phosphate and hydroxyapatite, which are specific to bone tissue. Secondly, they can be easily labeled with ^166^Ho^10^. Furthermore, the combination of ^166^Ho with EDTMP and DOTMP demonstrates high therapeutic efficiency in managing bone pain associated with metastatic bone disease^21^.

In terms of acute toxicity, we administered a dose of 37 MBq per mouse corresponds to a 117.8-fold increase over the minimum clinical dose (1.1 GBq/70 kg patient) used in prior studies^15^, which is consistent with the ICH M3(R2) guidelines for preclinical toxicology requiring high-dose safety margins. The ligands were used at 205 mg/kg (EDTMP) and 387 mg/kg (DOTMP), which remain within safe limits to 12.3-fold the clinical dose for NOAEL (No Observed Adverse Effect Level) in animals^27^. These doses did not lead to significant abnormalities in health, body weight, hematological or biochemical parameters, or severe organ damage. Minor changes, such as decreased platelet counts in the ^166^Ho-EDTMP group, increased creatinine levels, and variations in GOT and GPT levels, were noted in isolated cases and are expected to recover over time, similar to observations with ^153^Sm-EDTMP^16^. Histopathological analysis revealed mild chronic hepatitis and interstitial nephritis in the 14-day group, which is consistent with studies involving beta and alpha radionuclides such as ^153^Sm, ^177^Lu, ^223^Ra and ^211^At^16,18,42^. These findings align with the NOAEL criteria for safety pharmacology studies^42^.

During the 30-day observation period at therapeutic doses (18.5, 37, and 74 MBq/kg), no signs of bone marrow toxicity or health deterioration were observed in ICR mice. The doses used in the experiment were based on previous studies, such as 37 MBq/kg for ^153^Sm-EDTMP^30^ and ^177^Lu-EDTMP and 22.2 MBq/kg for ^166^Ho-DOTMP (Bayouth et al.)^14^. In our proposed therapeutic scheme, an activity of 18.5 MBq/kg, approximately half of the standard dose used for ^153^Sm-EDTMP, is expected to support the potential of ^166^Ho as a viable alternative for bone pain therapy. Compared with those treated with ^166^Ho-DOTMP, the weight gain of the mice treated with ^166^Ho-EDTMP was greater, suggesting a more favorable safety profile. All the treated groups presented a slight reduction in relative spleen weight, reflecting immune cell activity in response to the radiopharmaceuticals. Clinical studies with ^153^Sm-EDTMP have been reported to cause transient decreases in WBC (91–131 × 10^9^/L) and hemoglobin (8.5–8.9 g/dL) during weeks 2–4 at 3.7–37 MBq/kg^30,31^. In contract, ^166^Ho-EDTMP and ^166^Ho-DOTMP maintained WBC (3.6–8.5 × 10^9^/L), hemoglobin (12.5–15.1 g/dL), and PLT (545–787 × 10^9^/L) within normal limits, confirming good systemic safety.

Histological analysis revealed reduced osteocytes and osteoblasts in the femurs and sternums, likely due to the effects of beta-irradiation on bone tissue^43^. At doses of 37–74 MBq/kg, these findings indicate mild and localized bone responses without irreversible damage. However, bone regeneration and proliferation were observed, indicating recovery. It is reasonable that the biological half-life of ^166^Ho-DOTMP is approximately 44 h, with a sevenfold greater concentration in trabecular bone than in cortical bone, which could explain the localized effects of radiation^14^.

Interestingly, bone marrow analysis revealed that large, round cells constituted approximately 30% of the marrow at a dose of 74 MBq/kg for ^166^Ho-EDTMP, whereas they constituted approximately 20% for ^166^Ho-DOTMP at the same dose. This difference is attributed to the absorption of ^166^Ho-EDTMP in the bone marrow of ^166^Ho-EDTMP being 3.47 times greater than that of ^166^Ho-DOTMP, as reported by Pedraza-Lopez et al.^16^. At 18.5 MBq/kg, the density of these cells was approximately 10%. The increase in dose correlated with a greater density of large cells, likely due to increased radiation absorption and marrow cell response^43^. Treatment of human multiple myeloma with ^166^Ho-DOTMP showed that the absorbed dose to red bone marrow and bone surface were 0.517 and 0.920 mGy/MBq, respectively (56%). In comparation, ^153^Sm-EDTMP injection, the absorbed dose ratio between red bone marrow and trabecular bone was 46% (1.86 and 2.32 mGy/MBq, respectively), while FDA data reported 1.54 and 6.76 mGy/MBq (23%), respectively^12,30,46^. The observed cells included megakaryocytes, lymphoblasts, and myeloblasts, their progenitors of lymphocytes, hematopoietic stem cells, progenitor cells and macrophages, which revealed that active hematopoiesis and hematopoietic precursors and megakaryocytes were preserved^33^. This may be explained by the significant fluctuation in PLT reduction in the high-dose 74 MBq/kg ^166^Ho-DOTMP injection group, as well as inflammatory rupture and the restoration of blood cells following the reduction processes in the early stages after radiopharmaceutical injection. This is also possibly due to radiolabeled compounds inducing molecular changes in the bone marrow microenvironment^36^. The drawbacks can be mitigated by leveraging the fact that bone marrow is not the dose-limiting organ, allowing repeated administrations at lower doses compared to ^153^Sm. Moreover, treatment with ^166^Ho has been shown to permit rapid recovery of both lymphoid and myeloid cell lineages^38^.

Importantly, no signs of abnormal proliferation, clustering, or tumors were detected, and no significant reductions in WBC, red blood cell (RBC), or PLT counts were detected. While H&E staining provided valuable insights, additional diagnostic methods, such as immunohistochemistry, flow cytometry, cytological analysis, genetic testing, or special staining, are recommended for more precise identification of cell types and abnormalities^32^. In summary, histopathological findings confirmed mild, localized bone and marrow changes. ^166^Ho-DOTMP exhibited greater stability, less osteoblastic fibrosis, and better marrow preservation than ^166^Ho-EDTMP, supporting its potential as the preferred therapeutic candidate.

## Methods

### Ethics declarations

This study was conducted in accordance with the ARRIVE guidelines. All animal care and experimental protocols were reviewed and approved by the animal ethics committee of the Dalat Nuclear Research Institute and complied with the relevant guidelines for the care and use of animals. Animal experiments were performed following internationally recognized standards. The subjects were male and female ICR mice, aged 6–7 weeks, with albino coats sourced from the Ho Chi Minh City Biotechnology Center, Vietnam. The mice were housed in an environment with filtered air and maintained at a temperature of 22.05 ± 2.97 °C, a relative humidity of 55 ± 20%, and a 12-hour light/dark cycle (lights on at 8:00 a.m. and off at 8:00 p.m.). Food and water were provided according to the supplier’s guidelines.

### Preparation of ^166^Ho, ^166^Ho-DOTMP, and ^166^Ho-DOTMP

^166^Ho was prepared in a custom nuclear reactor via a thermal neutron capture reaction ^165^Ho(n,γ)^166^Ho at a thermal neutron flux of 2.3 × 10^13^ n.cm^− 2^.s^− 1^ for a period of 100–132 h. To produce ^166^Ho, 200 mg of natural ^165^Ho (100% abundance, Sigma Aldrich, USA) in oxide form was irradiated, followed by a cooling period of 24 h. The irradiated ^165^Ho_2_O_3_ was then dissolved in 4.0 mL of 0.05 N HCl to obtain a ^166^HoCl_3_ solution^10,13,21^. The radioisotope ^166^Ho was identified using high-purity germanium (HPGe) gamma spectrometry (Dspec GC1518, Canberra, USA). The radioactivity of the solution was measured (Capintec, USA), and radiochemical purity was tested using paper chromatography (PC) (Advantec, Japan) on Whatman No.1 paper as the stationary phase using 10 mM DTPA (pH 4) and 10% ammonium acetate: methanol (1:1, v/v) as mobile phase. Radioactivity distribution on the chromatograms was analyzed using a radioautography system (Cyclone, PerkinElmer).To prepare ^166^Ho-EDTMP, as described previously^10,21^ with optimized results, 336 mg of EDTMP (TCI, Japan) was dissolved in 1.1 mL of 2 N NaOH, and distilled water was added to a final volume of 2.918 mL (pH 7.0, concentration 115.13 mg/mL or 0.264 mM/mL). Then, 146.0 µL of ^166^HoCl_3_ solution (3.025 MBq, ~ 6.371 mg Ho^3+^, 0.264 mM) was added, resulting in a 1:20 molar ratio. The mixture was incubated for 15 min at 24 °C. The radiochemical purity of ^166^Ho-EDTMP was tested via a PC using Whatman No. 1 paper as the stationary phase and ammonia: methanol: water (1:10:20, v/v/v) as the mobile phase, and the product was filtered through a 0.2 μm sterile filter (Sartorius, Germany). Similarly, to prepare ^166^Ho-DOTMP, 634 mg of DOTMP (BLD Pharma, China) was dissolved in 2.5 mL of 2 N NaOH, and distilled water was added to a final volume of 4.380 mL (pH 7.0). Then, 146.0 µL of ^166^HoCl_3_ solution was added, achieving a 1:30 molar ratio. The radiochemical purity of ^166^Ho-DOTMP was also tested using a PC^10,22^.

### Acute toxicity tests

Acute toxicity testing was conducted in accordance with the single-dose toxicity test method outlined by the European Medicines Agency for the application of therapeutic radiopharmaceuticals^16^. The in vivo toxicology tests were performed in compliance with Good Laboratory Practice. Typically, 90 mice (45 males and 45 females, with a mean weight of 19.21 ± 1.99 g) were divided into three equal groups and fasted for 3 h prior to the experiment. The groups consisted of 30 saline-injected mice, 30 mice injected with ^166^Ho-EDTMP, and 30 mice injected with ^166^Ho-DOTMP. Each mouse received a dose of 100 µl of ^166^Ho-EDTMP or ^166^Ho-DOTMP (1850 MBq/kg; 37 MBq ^166^Ho; 4.10 mg of EDTMP or 7.75 mg of DOTMP) via tail vein injection. At 24 h postinjection, 10 animals of each sex were randomly selected for analysis, while the remaining 5 animals of each sex were assessed after 14 days. The body weights of the mice were measured. The mice were euthanized via a combination of ketamine and xylazine. Blood samples were collected for hematological analysis using a hematology analyzer (XN-1000, Sysmex, Japan) and for biochemical analysis via a biochemistry analyzer (AU 680, Beckman Coulter, Japan). Liver, kidney, spleen, and femur tissues were preserved in 10% neutral buffered formalin for histopathological examination^32,43^.

### Safety assessment of mice injected with ^166^Ho-EDTMP and ^166^Ho-DOTMP

We conducted a safety assessment on 70 male and female mice, with a mean weight of 20.37 ± 3.01 g, which were divided into seven groups of 10 mice each (5 males and 5 females). The intravenous injection consisted of seven doses, including 6 doses of 100 µl per mouse of 370 kBq ^166^Ho-EDTMP/^166^Ho-DOTMP (41.07 µg EDTMP/77.54 µg DOTMP), 740 kBq ^166^Ho-EDTMP/^166^Ho-DOTMP (82.14 µg EDTMP/155.08 µg DOTMP) and 1480 kBq ^166^Ho-EDTMP/^166^Ho-DOTMP (164.28 µg EDTMP/310.16 µg DOTMP), alongside one group receiving 100 µl of 0.9% saline per mouse.

The general health and body weight of the mice were monitored over 30 days. At the end of the study, the mice were euthanized using ketamine and xylazine. Blood samples were collected via cardiac puncture and collected in EDTA tubes for analysis. The hematological and biochemical parameters measured included RBC, WBC, PLT, HCT, HGB, GOT, and GPT^43,44^. Tissue samples such as liver, kidney, spleen, sternum, and femur samples were collected, preserved in 10% neutral buffered formalin, and embedded in paraffin blocks. The liver was collected from the largest lobes, and the spleen and kidneys were collected as whole organs. The femur included the articular cartilage, epiphysis, physis, and marrow cavity. Femurs and sternums were cleaned of muscles, ligaments, and tendons and then decalcified and embedded in paraffin. The longitudinally trimmed femoral and sternal samples were sectioned at 4.0 μm, stained with H&E, and examined under light microscopy magnification.

Bone lesions were observed according to the International Harmonization of Nomenclature and Diagnostic Criteria for Lesions in Rats and Mice criteria^32^. Histopathological diagnostics were conducted on the basis of expert observations, and the results were compared with those of controls. Histological observations were graded via a five-point scale^45^.

### Statistical analysis

The results are presented as the means ± SDs or means ± SEMs, and a P value < 0.05 was considered significant. Two-way ANOVA, followed by Tukey’s or Sidak’s multiple comparisons tests, was used to analyze body weight and weight gain data. Student’s t test was used to estimate differences in body weight and hematological and biochemical parameters. Chromatograms were analyzed using OptiQuant 5 software.

## Conclusion

Our findings demonstrated that both ^166^Ho-EDTMP and ^166^Ho-DOTMP provide important data on acute radiotoxicity at high doses, particularly in bone tissue and bone marrow radiotoxicity observations at therapeutic doses. At a dose of 3.7 MBq per mouse, no acute toxicity was observed after 14 days of follow-up. Additionally, at doses ranging from 0.37 to 1.48 MBq per mouse, no indications of bone marrow toxicity or significant adverse effects were noted in ICR mice over 30 days. Notably, an increase in active hematopoiesis was observed in the bone marrow. While these results suggest minimal radiotoxicity, further studies are needed to optimize the formulation and assess long-term effects in both preclinical and clinical settings, particularly regarding pain relief in patients with bone metastases, where ^166^Ho may serve as an appropriate alternative palliative option.

## Supplementary Information

Below is the link to the electronic supplementary material.

Supplementary Material 1

## Data Availability

The datasets generated and analyzed during the current study are available from the corresponding author on reasonable request.
