# Supplementary material for: Acute radiotoxicity studies and safety assessment of 166Ho-EDTMP and 166Ho-DOTMP for the palliative treatment of bone metastases
Source: Sci Rep. 2025 Nov 27;15:42305. doi: 10.1038/s41598-025-26098-7 (PMC12661045; doi:10.1038/s41598-025-26098-7)
Supplement: Supplementary file 1 — Supplementary Material 1 [file 41598_2025_26098_MOESM1_ESM.docx]

**SUPPLEMENTARY INFORMATION FOR**

**ACUTE RADIOTOXICITY STUDIES AND SAFETY ASSESSMENT OF ^166^Ho-EDTMP AND ^166^Ho-DOTMP FOR THE PALLIATIVE TREATMENT OF BONE METASTASES**

Ho Hong Quang Dang^1^, Thi Khanh Giang Nguyen^1^, Thi Ngoc Nguyen^1^, Thanh Binh Nguyen^1^, Ngoc Bao Nam Dinh^1^, Ngoc Dieu Thao Le^2^, Thu Minh Chau Nguyen^3^, Hong Ngoc Quy Dang^1^, Van Tien Le^4^, Thanh Nhan Nguyen^1^, Thi Thu Nguyen^1^*

^1^Center for Research and Production of Radioisotopes, Nuclear Research Institute, Dalat City, Vietnam

^2^Department of Anapathology, Cho Ray Hospital, Ho Chi Minh City, Vietnam

^3^Hanoi Medical University, Vietnam

^4^Lam Dong General Hospital, Dalat City, Vietnam

**Supplementary information:**

**1. Supplementary methods S1**

Factors affecting labeling efficacy, including molar ratio, pH, reaction time and temperature, were investigated to determine the optimal radiolabeling conditions.

**Supplementary results S1**

The results of ^166^Ho-EDTMP and ^166^DOTMP conjugation formations are presented in **Table S1.**

The chosen radiolabeling concentrations was based on molar ratios (moles of ^166^Ho to moles of EDTMP/DOTMP) of 1:20 for ^166^Ho-DOTMP and 1:30 for ^166^Ho-DOTMP. The optimal pH was determined to be ~7 for both radiolabeling procedures. A reaction time of 15 minutes at a temperature of 24 °C was used under our experimental conditions.

| **Molar ratios**  ^166^Ho-EDTMP eff.^*^ (%)  ^166^Ho-DOTMP eff. (%) | **1:1**  93.75 ± 1.62  3.66 ± 0.49 | **1:10**  99.60 ± 0.42  32.93 ± 1.91 | **1:20**  99.71± 0,20  84.49 ± 1.34 | **1:30**  99.49± 0.46  99.20 ± 0.30 |
| --- | --- | --- | --- | --- |
| **pH**  ^166^Ho-EDTMP eff. (%)  ^166^Ho-DOTMP eff. (%) | **5**  99.67 ± 0.12  99.59 ± 0.11 | **6**  99.69 ± 0.14  99.69 ± 0.18 | **7**  98.80± 0.62  99.63 ± 0.25 | **8**  96.59± 0.76  99.75 ± 0.09 |
| **Reaction time (min)**  ^166^Ho-EDTMP eff. (%)  ^166^Ho-DOTMP eff. (%) | **5**  99.85 ± 0.11  98.65 ± 0.49 | **15**  99.75 ± 0.36  98.95 ± 0.21 | **30**  99.55 ± 1.11  99.40 ± 0.14 | **45**  99.70± 0.19  99.65 ± 0.07 |
| **Temperature (℃)**  ^166^Ho-EDTMP eff. (%)  ^166^Ho-DOTMP eff. (%) | **4**  99.71 ± 0.22  98.65 ± 0.49 | **24**  99.49 ± 0.27  99.65 ± 0.21 | **37**  99.45 ± 0.35  99.35 ± 0.49 | **50**  99.40± 0.22  99.30 ± 0.14 |

**Table S1.** Results of effect on radiolabeling efficacy of ^166^Ho-EDTMP and ^166^Ho-DOTMP preparation (n = 3). *Note:* Results are expressed as mean ± standard deviation; n = 3 for each experiment point (eff.: Efficacy). The Labeling efficiency is analyzed by paper chromatography in ammonia: methanol: water (1:10:20, v/v ratios), 120 minutes. Results were calculated on a Cyclone radiograph and processed with OptiQuant 5.0 software.

**Supplementary conclusions S1**

The results show that the optimal labeling efficiency for the preparations of ^166^Ho-EDTMP and ^166^DOTMP are achieved at > 98%.

**2. Supplementary methods S2**

The radiochemical purity of ^166^Ho was determined using paper chromatography. The chromatography strip was placed in a tank containing a solvent mixture of 10% ammonium acetate and methanol (1:1), or 10 mM DTPA (pH 4). The strip was developed until the solvent front reached the solvent front line. Afterward, the strip was removed and analyzed using a storage phosphor screen. Chromatograms were processed using OptiQuant 5 software.

The stability of ^166^Ho-EDTMP and ^166^Ho-DOTMP was evaluated in 0.9% NaCl and 0.02 M PBS after storage at 4 °C and 24 °C for 3 days. Additionally, the samples were incubated in 0.05% human serum albumin (HSA) at 37 °C for 3 days. Radiochemical purity was assessed every 24 hours using paper chromatography.

**Supplementary results S2**

The radiochemical purity of ^166^Ho was determined to be >99.9%. In the solvent mixture of 10% ammonium acetate and methanol, ^166^Ho remained at the origin (Rf = 0.0–0.25), as shown in **Fig. S1a**. In the 10 mM DTPA solvent, ^166^Ho migrated to the front (Rf = 0.9–1.0), as shown in **Fig. S1b.**

The stability of ^166^Ho-EDTMP (**Fig. S1c**) and ^166^Ho-DOTMP (**Fig. S1d**) in 0.9% NaCl and 0.02 M PBS after 3 days of storage at 4 °C and 24 °C was assessed, with radiochemical purity values exceeding 96% (P = 0.2743 and P = 0.7196, ordinary one-way ANOVA followed by Brown-Forsythe analysis, Fig. S1c and Fig. S1d). This indicates no significant differences in radiochemical purity under these conditions. However, a significant difference was observed between 24 °C in 0.9% NaCl and 24 °C in 0.02 M PBS for ^166^Ho-EDTMP stability (*P = 0.038, two-way ANOVA with Tukey's multiple comparisons test), but the radiochemical purity remained above 95%.

Furthermore, after incubation at 37 °C for 3 days in 0.05% human serum albumin, the radiochemical purity of ^166^Ho-EDTMP and ^166^Ho-DOTMP was 97.10 ± 0.14% and 96.85 ± 0.63%, respectively, which are shown in **Fig. S1c** and **Fig. S1d**.


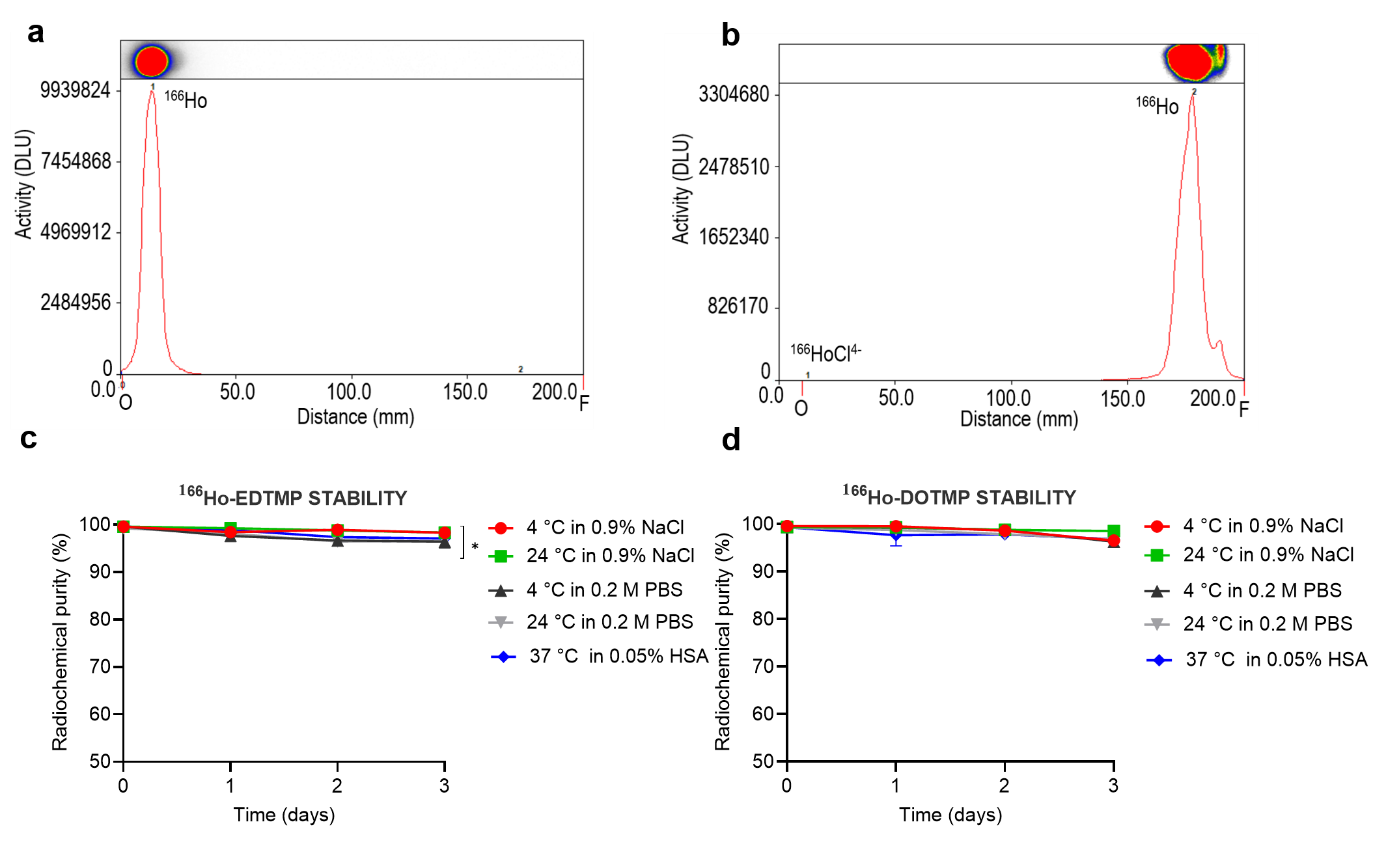


**Figure S1**. Radiochemical purity of ^166^Ho and stability of ^166^Ho-EDTMP and ^166^DOTMP. The experiments were performed in duplicate and repeated three independent times (n=3). (**a**) Radiochemical purity of ^166^Ho using PC in 10% ammonium acetate: methanol (1:1). (**b**) Radiochemical purity of ^166^Ho using PC on Whatman No.1 paper as the stationary phase using 10 mM DTPA solvent as mobile phase. (**c**) Stability of ^166^Ho-EDTMP. (**d**) Stability of ^166^Ho-DOTMP.

**Supplementary conclusions S2**

The results indicate no significant differences in the stability studies of ^166^Ho-EDTMP and ^166^DOTMP. Radiochemical purity remained above 95% at 4 °C and 24 °C in 0.9% NaCl and 0.2 M PBS, as well as at 37 °C in 0.05% HSA after 3 days of storages.

**3. Supplementary results S3**

Body weight measurements and body weight gain (in grams) were recorded for the mice in **Table S2**.

| **Group I**  **(n=10)** | **Control** | | **^166^Ho-EDTMP** | | **^166^Ho-DOTMP** | |
| --- | --- | --- | --- | --- | --- | --- |
|  | **Male** | **Female** | **Male** | **Female** | **Male** | **Female** |
| **Day 0** | 20.45 ± 2.14 | 17.96 ± 1.80 | 19.94 ± 1.64 | 19.33 ± 1.67 | 19.43 ± 2.10 | 18.20 ± 1.68 |
| **Day 1** | 20.76 ± 2.28 | 18.18 ± 1.68 | 20.23 ± 1.66 | 19.57 ± 1.76 | 19.73 ± 1.98 | 18.46 ± 1.71 |
| **change** | 0.31 ± 0.14 | 0.22 ± 0.13 | 0.29 ± 0.11 | 0.24 ± 0.12 | 0.30 ± 0.14 | 0.26 ± 0.13 |
| **Group II (n=4-5)** | | | | | | |
| **Day 0** | 21.40 ± 2.68 | 18.40 ± 1.95 | 21.30 ± 2.71 | 20.00 ± 1.45 | 19.04 ± 1.92 | 18.32 ± 2.15 |
| **Day 14** | 25.98 ± 2.22 | 22.60 ± 1.39 | 25.25 ± 1.54 | 23.73 ± 2.88 | 23.10 ± 1.81 | 22.48 ± 2.03 |
| **change** | 4.58 ± 1.33 | 4.20 ± 1.33 | 3.95 ± 1.41 | 3.72 ± 1.41 | 4.06 ± 1.41 | 4.15 ± 1.41 |
|  | ^**^P=0.008 | ^*^P=0.0178 | ^*^P=0.0461 | P=0.0686 | ^*^P=0.0377 | ^*^P=0.0316 |

**Table S2.** The data are presented as the means ± SDs/SEMs (n=10) for the 0.9% NaCl and ^166^Ho-EDTMP or ^166^Ho-DOTMP groups on Day 1 and n = 4–5 for the Day 14 group. Each cell mean was compared with the other cell means in that column via two-way ANOVA with Sidak’s multiple comparisons test.

**4. Supplementary results S4**

Comparative data with ^153^Sm-EDTMP are presented in **Table S3**.

| **Parameters** | **^166^Ho-EDTMP/**  **^166^Ho-DOTMP** | **^153^Sm-EDTMP (Lexidronam)** | **References** |
| --- | --- | --- | --- |
| Radionuclide physical properties | 1.85 MeV (51%) 1.77 MeV (48%) E_ꞵmean_ 0.66 MeV  T_1/2_^:^ 26.8 hours  β-particle range:  Max.: 10.2 mm Mean: 2.2 mm | 0.808 MeV (18%), 0.705 MeV (50%,) E_ꞵmean_ 0.22 MeV  T_1/2_: 46.3 hours  β-particle range:  Max.: ~3 mm  Mean: 0.8 mm | Tan 2020; Klassen 2019; Dash et al., 2025 |
| WBC (× 10⁹/L) | From 3.64 ± 1.15 to 8.48 ± 1.24 at 14 and 30 days.  Normal range  2-10 (Mouse) | 6.28 ± 1.49; 6.15 ± 1.76 and 6.78 ± 1.75 at 3, 6 and 8 wk.  Normal range  4-10 (Human) | Sharma, *Frontier* 2017; FDA 2017;  en.wikivet.net;  Charles River 2020 |
| Hemoglobin (g/dL) | From 12.56 ± 1.76 to 15.13 ± 0.15. Normal range  10-16 (Mouse) | 8.9 ± 0.89, 8.5 ± 2.11 and 8.8 ± 2.15 at 3, 6 and 8 wk. Normal range 12-17.5 (Human) | Sharma, *Frontier* 2017; en.wikivet.net  WHO 2011 |
| PLT (× 10⁹/L) | From 545 ± 124 to 787 ± 95  Normal range:  160-410 (Mouse) | 113 ± 29; 91 ± 25 and 131 ± 46 at 3, 6 and 8 wk. Normal range 150-450 (Human) | Sharma, 2017; en.wikivet.net  Karyn E 2015, WHO 2011; FDA 2017 |
| GOT (U/L) | 126.50 ± 28.76 300.33 ± 225 Normal range:  54-298 (Mouse) | Data not found;  Normal range  7-56 (Human) | en.wikivet.net;  Sartor 2004; FDA 2017 |
| GPT (U/L) | 46.60 ± 10.60 to 80.67 ± 46.36; Normal range:  17-77 (Mouse) | Data not found; Normal range  8-48 (Human) | en.wikivet.net; Sartor 2004; WHO 2011; FDA 2017 |
| Creatinin (µM/L) | 18.50 ± 4.80 to 31.00 ± 1.73; Normal range:  17.6-79.5 (Mouse) | Data not found; Normal range:  53-115 (Human) | en.wikivet.net  WHO 2011; FDA 2017; Sartor 2004  ema.europa.eu |

**Table S3.** Summarizes the hematological and biochemical results obtained in our study at a dose of 18.5 MBq/kg (after 30 days) and at a higher dose of 1850 MBq/kg (after 14 days) treatment with ^166^Ho-EDTMP and ^166^Ho-DOTMP, comparative with ^153^Sm-EDTMP.

**5. Supplementary results S5**

Minimal to mild depletion of osteoclasts was observed in the femurs (23/58 mice, 39.6%) and sternums (12/58 mice, 20.6%) of groups injected with 18.5, 37, and 74 MBq/kg of ^166^Ho-EDTMP and ^166^Ho-DOTMP. The minimal levels of osseous lesion were observed in the femurs (5/58 mice, 8.6%) and sternums (1/58 mice, 1,7%). These findings are summarized in **Table S4**.

| **Group**  **(MBq/kg)** | **Control** | | **^166^Ho-EDTMP** | | | | | | **^166^Ho-DOTMP** | | | | | |
| --- | --- | --- | --- | --- | --- | --- | --- | --- | --- | --- | --- | --- | --- | --- |
|  | **0** | | **18.5** | | **37** | | **74** | | **18.5** | | **37** | | **74** | |
| **(n=5)** | **M** | **F** | **M** | **F** | **M** | **F** | **M** | **F** | **M** | **F** | **M** | **F** | **M** | **F** |
| **Femurs** |  |  |  |  |  |  |  |  |  |  |  |  |  |  |
| **Osteocytes and Osteoblasts** | | | | | | | | | | | | | | |
| *minimal* | - | - | 1 | 1 | 2 | 1 | 1 | 2 | 1 | 1 | 2 | 2 | - | 1 |
| *mild* | - | - | - | - | - | 1 | 1 | 1 | - | - | - | 1 | - | 1 |
| *moderate* | - | - | - | - | - |  | 1 | 1 | - | - | - | - | - | 1 |
| **Osseous lesion** | | |  |  |  |  |  |  |  |  |  |  |  |  |
| *minimal* | - | - | - | - | - | 1 | - | 1 | - | - | - | - | 1 | - |
| *mild* | - | - | - | - | - | - | - | 1 | - | - | - | - | - | 1 |
| **Sternums** |  |  |  |  |  |  |  |  |  |  |  |  |  |  |
| **Osteocytes and Osteoblasts** | | | | | | | | | | | | | | |
| *minimal* | - | - | - | 1 | 1 | - | 1 | 2 | - | 1 | 2 | 2 | - | 1 |
| *mild* | - | - | - | - | - | - | - | - | - | - | 1 | - | - | - |
| **Osseous lesion** | |  |  |  |  |  |  |  |  |  |  |  |  |  |
| *minimal* | - | - | - | - | - | - | - | - | - | 1 | - | - | - | - |

**Table S4.** Estimates of osteocyte and osteoblast depletion, along with fibro-osseous lesions in mice injected with ^166^Ho-EDTMP and ^166^Ho-DOTMP. M: Male, F: Female, Control Group: Injected with 0.9% NaCl.

**6. Supplementary S6:** Histopathological analysis of sternal bone marrow in mice injected with ^166^Ho-EDTMP and ^166^Ho-DOTMP revealed normal architecture of hematopoietic tissue. We present in the **Figure S2**:


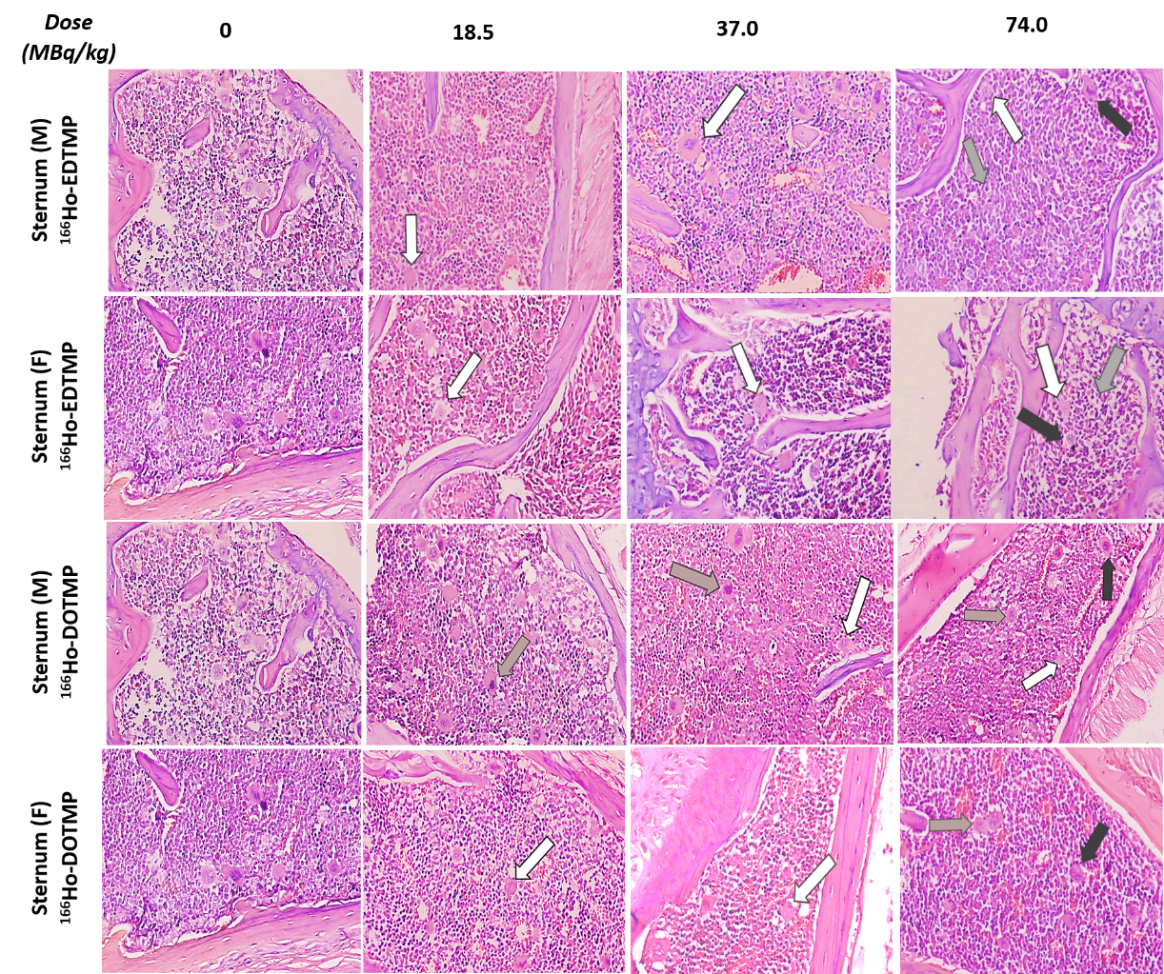


**Figure S2.** Representative histopathology images of sternal bone marrow (H&E staining, 400×). White arrow: Megakaryocyte, a large hematopoietic cell characterized by a multilobed nucleus. Gray arrow: Lymphoblast cell, identified by its 1–2 nucleoli, scant cytoplasm, and round or slightly irregular nucleus. Black arrow: Myeloblast cells, large hematopoietic precursor cells with scant cytoplasm, round or slightly irregular nuclei, fine chromatin, and 3–5 nucleoli.

Megakaryocytes, myeloblasts, and lymphoblasts were clearly identified and well preserved, with no evidence of necrosis, fibrosis, or abnormal cellular proliferation.

**7. Supplementary discussion S7**

Although ^166^Ho emits higher-energy β⁻ particles (Emax = 1.85 MeV) than other therapeutic radionuclides such as ^153^Sm, ^89^Sr, and ^177^Lu, its short half-life (26.8 h) limits systemic exposure while maintaining strong local irradiation to bone lesions. The moderate β⁻ penetration compare with ^153^Sm, ^89^Sr, and ^177^Lu, enables effective cross-fire irradiation of irregular or poorly perfused skeletal metastases. A concise comparison of the bone pain palliation radionuclides of interest, ^166^Ho, ^153^Sm, ^89^Sr, and ^177^Lu, is summarized in the following **Table S5**:

| **Characteristics** | **^166^Ho** | **^153^Sm** | **^89^Sr** | **^177^Lu** |
| --- | --- | --- | --- | --- |
| T_1/2_ (days) | 1.1 | 1.95 | 50.5 | 6.7 |
| E_ꞵmax_ (MeV) | 1.85 | 0.81 | 1.46 | 0.49 |
| E_ꞵmean_ (MeV) | 0.66 | 0.22 | 0.58 | 0.13 |
| β-particle range in tissue (mm) | Max.: 10.2 Mean: 2.2 | Max.: ~3.0 Mean: 0.8 | Max.: 8.0 Mean: 2.4 | Max.: 2.0 Mean: 1.8 |
| Diagnostic dose (MBq) | 740 -1,100 | 37-74 | 37 | 37-74 |
| Treatment dose (MBq) | 740 -1,100 18,500-74,000 | 1,850 | 148 | 1,850-3,700 |
| Bone surface mGy/MBq | 2.0-2.7(*)  0.92 (**) | 6.76 | 17 | 2.3-2.8 |
| Red marrow mGy/MBq | 0.4-0.9 (*)  0.51 (**) | 1.54 | 11 | 0.6-0.9 |
| Pain response rates (%) | 35 - 91% (^#^) | 62–74% | 60–95% | 77–95% |
| Red marrow (bone marrow ablation, Gy, repeat inject) | (20-60 Gy)  18.5-148 GBq  Used 18,500-74,000 MBq | 22 Gy | 11 | - |

**Table S5.** Comparison of physical characteristics of β⁻ for bone pain palliation agents.

*Notes: Sr-89: ICRP 53 (1987); ^89^Sr (2013) GE Healthcare reference ID: 3426437; EMA SPC, Sm-153: EMA SPC (2017); FDA label; Rajendran et al., J Nucl Med 1998, Lu-177: Dash and Das (2025); IAEA-TECDOC-1845*

(*) *Ho-166: Bayouth et al. (1995);* (**) *(Breitz et al. (2006); Bagheri et al. (2021) (^#^) Breits et al (2004)*
